# Effect of *Smilax* spp. and *Phellinus linteus* combination on cytotoxicity and cell proliferation of breast cancer cells

**DOI:** 10.1186/s12906-023-04003-x

**Published:** 2023-06-01

**Authors:** Kanwalat Chalertpet, Thanawitch Sangkheereeput, Prakaithip Somjit, Wichanee Bankeeree, Pattamawadee Yanatatsaneejit

**Affiliations:** 1 Department of Botany, Faculty of Science, Human Genetics Research Group, Chulalongkorn University, Bangkok, 10330 Thailand; 2 Department of Botany, Faculty of Science, Plant Biomass Utilization Research Unit, Chulalongkorn University, Bangkok, 10330 Thailand

**Keywords:** Herbal medicine, *Phellinus linteus*, *Smilax corbularia*, *Smilax glabra*, Breast cancer, Adjuvant drug

## Abstract

**Background:**

Although the prevalence of breast cancer (BC) has been reduced in recent years, proficient therapeutic regimens should be further investigated with the aim of further reducing the mortality rate. To obtain more effective treatment, the present study aimed to observe the effects of PL synergistically combined with *Smilax corbularia* and *S. glabra* extracts (PSS) on BC cell lines, MCF7, T47D, MDA-MB-231, and MDA-MB-468.

**Methods:**

The half-maximal inhibition (IC_50_) concentrations of PSS and PL were determined in a dose- and time-dependent manner using MTT assay. The activity of PSS and PL on anti-BC proliferation was evaluated using BrdU assay, and colony formation assay. Moreover, cell cycle analysis and apoptosis induction as a result of PSS and PL exposure were investigated using propidium iodide (PI) staining and co-staining of annexin V DY634 and PI combined flow cytometric analysis, respectively. Finally, changes in the mRNA expression of genes involved in proliferative and apoptotic pathways (*MKI67*, *HER2, EGFR, MDM2, TNFα, PI3KCA, KRAS, BAX, and CASP8*) were explored using RT-qPCR following PSS and PL treatment.

**Results:**

The PSS and PL extracts exhibited significant potential in BC cytotoxicity which were in were in dose- and time-dependent response. This inhibition of cell growth was due to the suppression of cell proliferation, the cell cycle arrest, and the induction of apoptosis. Additionally, an investigation of the underlying molecular mechanism revealed that PSS and PL are involved in downregulation of the *MKI67, HER2*, *EGFR*, *MDM2*, *TNFα, and PI3KCA* expression.

**Conclusions:**

This present study has suggested that PSS and PL possess anti-BC proliferative activity mediated via the downregulation of genes participating in the relevant pathways. PSS or PL may be combined with other agents to alleviate the adverse side effects resulted from conventional chemotherapeutic drugs.

**Supplementary Information:**

The online version contains supplementary material available at 10.1186/s12906-023-04003-x.

## Background

Despite the advancements that have been made in health technology, the incidence of cancer and mortality globally continues to grow, attributing up to 47% over the last year. Breast cancer (BC) was ranked first in terms of cancer incidence in the majority of countries, and was the second leading cause of death in woman worldwide [1]; therefore, evolving strategies of BC cancer treatment and prevention need to be addressed. Currently, several alternative therapeutic procedures for patients with cancer are in use, including nanomedicine, immunotherapy, and stem cell transplantation [2, 3]. Not only have these approaches been applied, but herbal medicines have also been used as an adjuvant treatment, since they have been shown to reduce the adverse effects resulting from conventional treatments, alleviate symptom-associated diseases, increase the efficacy of treatment programs, and prevent cancer recurrence [4–8].

Medicinal herbs have been broadly studied in view of their different bioactive compounds and pharmacological actions [9]. *Phellinus*, a traditionally used and edible mushroom, has been extensively found and utilized in China, Japan, South Korea, and Thailand. *Phellinus linteus* (PL) has been shown to have a prominent role in anti-tumor activity, largely as a result of the main component polysaccharides [ß(1–3)-linked glucan chain with ß(1–6) branch points] [10, 11]. The first published study of PL extract as an anti-cancer compound appeared in 1968 [12]. Subsequently, a large number of studies have demonstrated and elucidated its immuno-modulator effects, including repression of cell metastasis and cell proliferation, and activation of apoptosis events against cancer both in vitro and in vivo [13–19].

*Smilax corbularia* (SC) and *Smilax glabra* (SG) are distributed throughout the eastern and south-eastern regions of Asia [20]. On account of their possession of phytochemicals, which are mainly from phenolic compounds [21], they have been shown to exhibit pharmacological activity, including immunomodulatory, anti-inflammatory, anti-microbial and anti-tumor properties [22–27]. Recently, they have been prescribed for the alleviation of symptoms associated with rheumatism, syphilis, diabetes and cancer [28–30]. Owing to the different major bioactive agent groups associated with an herbal extract compound of PL, SC and SG (denoted as PSS), this compound was taken to investigate if they have a synergistic anti-cancer effect in BC compared to PL together with the aim of enhancing their medicinal activities in order to change BC behavior with respect to cell proliferation and apoptosis.

The onset of BC is provoked by numerous factors, including physical activity, age, breast tissue density, family history, and genetic mutations. Approximately 25% of hereditary BC has been shown to result from mutations of BC susceptibility gene-1 (*BRCA1*), which increase the incidence of early-onset BC up to 80% [31]. *BRCA1* is thus used as BC tumor marker, as well as human epidermal growth factor receptor 2 (*HER2*) [32, 33]. In addition to *HER2*, epidermal growth factor receptor (*EGFR),* or *HER1,* is another oncogene in ErbB family, the overexpression of which is implicated in BC proliferation and driving metastasis, including reducin the survival rate [34, 35]. Together with the overexpression of mouse double minute 2 (*MDM2*), it has a role in BC via the augmentation of BC invasion and migration through negatively regulating P53 [36, 37] as well as tumor necrosis factor α (*TNFα*) which elicits inhibition of cell proliferation, induction of apoptosis, or even the enhancement of cell migration, as well as contributing to poor prognosis outcomes [38]. Moreover, the co-exist between *PI3KCA*, a catalytic subunit of PI3K protein, with the Kirsten ras oncogene (*KRAS*) mutation was identified in advanced grade carcinogenesis, together with *KRAS* gene silencing, which resulted in inactivation of *PI3K*CA pathway [39, 40]; *PI3KCA* and *KRAS* are therefore good candidate genes to study. Finally, avoidance of apoptosis is a typical characteristic of cancer, including BC [41]; therefore, BCL2-associated X-protein (*BAX*) and caspase-8 (*CASP8*), which participate in intrinsic and extrinsic apoptotic pathways [42, 43], merited further investigation. It was possible that a lower expression of *BAX* and *CASP8* may have led to an impairment of apoptotic cell death.

Taken together, the present study aimed to evaluate whether PSS could lead to an enhancement in anti-BC activity compared to PL, and the impact that they have on the BC-causative genes. As described, PSS and PL may be administered with chemotherapeutic agents to minimize the adverse side effects associated with conventional treatments, which should prolong the patients’ survival rate and contribute to an improved quality of life for the patients.

## Methods

### Cell culture

The BC cell lines used in the present study were MCF7, T47D, MDA-MB-231, and MDA-MB-468, which were purchased from American Type Culture Collection (ATCC, VA, USA). The cells were cultured in Gibco® Dulbecco’s modified Eagle’s medium (DMEM) (Thermo Fisher Scientific, MA, USA) supplemented with 10% Gibco® fetal bovine serum (FBS) (Thermo Fisher Scientific) and 1% Gibco® antibiotic–antimycotic (Thermo Fisher Scientific) and maintained in a humidified atmosphere with 5% CO_2_ at 37 °C.

### Preparation and characterization of extracts

Crude powder from fruiting body of (PL, rhizome of SC, and SG obtained from Herb for You co., Ltd. (Bangkok, Thailand) and Nature Herbs International Holding co., Ltd (Bangkok, Thailand). PL, SC, and SG were mixed at the ratio 3:1:1 to form PSS. The extraction of PSS and PL powders was conducted with distilled water at the solid-to-liquid ratio of 1:50 using a Soxhlet apparatus at 80 °C for 5 h. Then, the extracts were filtered using Whatman No. 1 filter paper and the filtered extracts were oven-dried at 70 °C overnight. The extraction yield was calculated according to the following formula (1):

1 $$\mathrm{Yield}\;(\mathrm{g}/\mathrm{g}) =\frac{gram\;of\;died\;extract\;obtained\;after\;evaporation}{gram\;of\;sample\;dry\;weight}$$

The total polysaccharide and total phenolic contents of crude extracts were determined by the Anthrone [44] and Folin-Ciocalteu’s [45] methods, respectively. The compound profiling from each crude extract (0.1 mg/mL in deionized water with 0.1% formic acid) was characterized using LC-QTOF-MS (Bruker Daltonik, Bremen, Germany). The chromatographic separation was accomplished using Acclaim RSLC 120 C18 column (2.2 μm, 100 × 2.1 mm) (Thermo Fisher Scientific, Dreieich, Germany) at 40 °C with a flow rate of 0.3 mL/min and the injection volume was 10 μl. The mobile gradient system started with 5% acetonitrile (ACN) and 95% water (0.1% formic acid), increasing to 20% ACN in 5 min, 40% ACN in 5 min, 80% ACN in 5 min, and 95% ACN until the run ended. The MS analyzer was operated in positive electrospray ionization mode (ESI +) with nebulizer gas pressure of 1.8 bar (N_2_), dry gas flow of 8.0 L/min, dry gas temperature of 220 °C, capillary voltage of 4500 V. TOF- MS were scanned over a range of m/z from 50 to 1300. The identification of compounds was carried out with Data Analysis 4.4 and TASQ 1.4 software packages (Bruker Daltonics Bremen, Germany) by comparing the retention time, peak intensity, and mass spectra with spectral libraries including Bruker MetaboBASE Personal Library, ESI–MS/MS Library, MS-DIAL Library, BMDMS-NP Library and ReSpect Library. The extract was kept in a refrigerator at 4 °C until further use.

### MTT assay

To determine half-maximal inhibitory concentration (IC50) over an optimal treatment period, MTT (Abcam, Cambridge, UK) assay was performed according to the manufacturer’s instructions. Briefly, cells were seeded in 96-well plates at a density of 5 × 10^3^ cells/well, and incubated at 37 °C for 24 h. The cells were then treated with the PSS and PL mixture at different concentrations between 0 – 250 µg/ml and 0 – 2,500 µg/ml, respectively, for 24,48, 72 and 96 h. Cisplatin (Sigma-aldrich) at concentrations of 0 – 40 µM was administered and used as a positive control. After that cultured medium was discarded, and MTT at a final concentration of 0.5 mg/ml was added and incubated for 150 min at 37 °C. After this period of incubation, the medium was removed, and 100 µl of DMSO was added to each well. Cell viability was measured at 492 and 630 nm using a microplate reader. Inhibition percentage compared between the untreated, and PSS-, PL- or cisplatin-treated groups were determined, and reported as percentages of the untreated control. Then, graphs were generated showing the inhibition percentages versus the log concentration of PSS, PL or cisplatin to calculate the IC_50_ values using GraphPad Prism, version 8 (GraphPad, CA, USA).

### BrdU incorporation assay

Cells were seeded at 5 × 10^3^ cells/well in a 96-well plate for 24 h prior to treatment. After 48 h of treatment with the PSS, PL or cisplatin following the IC_50_ concentrations (predetermined from the MTT assay), BrdU labelling solution was added according to the manufacturer’s instruction (Roche Diagnostics, Basel, Switzerland). After 24 h, the extent of cell proliferation was detected at 370 and 492 nm. Cell proliferation graphs were plotted using GraphPad Prism, version 8 (GraphPad) and reported as percentages of the control comparing between the untreated- and treated-group.

### Colony formation assay

To assess whether treatment with PSS, PL and cisplatin was able to inhibit BC cell survival, a colony formation assay with certain modifications was performed. Briefly, cells were seeded at 5 × 10^4^ cells/well in a 24-well plate a day before treatment. Then, the treatments were performed using the IC_50_ concentrations of PSS, PL, and cisplatin derived from the MTT assay for 3 days. Cells were collected by trypsinization, measured out at a density of 1 × 10^3^ cells/well, seeded in a 60 mm dish, and further cultured in a humidified atmosphere with 5% CO_2_ at 37 °C for 14 days with a change of culture media every 3 days. After that culture media had been discarded, cells were washed 2 times with PBS and stained using 1 ml of 0.5% crystal violet solution (Abcam) dissolved with 20% methanol (MilliporeSigma, MA, USA) for 30 min. Finally, cells were washed again 2times with PBS, and then dried at room temperature overnight, Images of the stained cells were taken using Azure™ c500 gel imaging system (Azure Biosystems, CA, USA). Colonies were counted using AzureSpot analysis software (Azure Biosystems), and the surviving fraction (SF) was calculated as previously described [46].

### Cell cycle analysis using propidium iodide (PI)

Flow cytometric analysis of the cell cycle with PI DNA staining was performed. First, cells were seeded at 5 × 10^5^ cells/well in a 6-well plate a day prior to treatment. Treatment of cells with PSS, PL, and cisplatin was subsequently performed using the IC_50_ concentrations of these compounds derived from the MTT assay for 3 days. Cells were then collected by trypsinization and washed 2 times with PBS. Cells were fixed with cold 70% ethanol (MilliporeSigma) at 4 °C overnight, and then were washed 2 times with PBS, before being centrifuged at 14,000 rpm for 15 min. Next, 50 µl of a 100 µg/ml stock of RNase (Sigma-aldrich) was added, followed by 5 µl of PI staining solution (BD Pharmingen, CA, USA), and the cells were incubated for 15 min at room temperature. Finally, the cells were subjected to flow cytometry analysis, and the percentages of cell populations in each phase of cell cycle were plotted using GraphPad Prism, version 8 (GraphPad).

### Annexin V apoptotic assay

An annexin V-DY-634/PI apoptosis detection kit (cat no. ab214484; Abcam) was used. After cells had been seeded and treated as described in the “cell cycle analysis using PI” section above, the cells were collected and washed 2 times with PBS. The cells were then stained with PI and DY-634 according to the manufacturer’s instructions. Subsequently, flow cytometry analysis was performed. Graphs showing the percentages of early apoptotic late apoptotic cells, and necrotic cells were generated using GraphPad Prism, version 8 (GraphPad).

### Gene expression analysis

After the cells had been treated with the PSS or PL extract, as described in the “Cell cycle analysis using PI” section, cells were collected by trypsinization. RNA extraction was performed using Invitrogen® Trizol™ (Thermo Fisher Scientific, CA, USA) according to the manufacturer’s protocol. Subsequently, 750 – 1000 ng of total RNA from each treated sample was used to synthesize cDNA using a cDNA synthesis kit (Biotechrabbit, Hennigsdorf, Germany), according to the manufacturer’s specification. The cDNA was used for RT-qPCR experiments using 4X CAPITAL™ qPCR Green Master Mix (Biotechrabbit) with forward (FW) and reverse (RW) candidate gene primers as shown in Table 1 (*GAPDH* was used as the internal control). The amplifications were performed using a QuanStudio™ 5 Real-time PCR System (Thermo Fisher Scientific) with the following thermocycling conditions: 40 cycles of 95 °C for 15 secs, 60 °C for 30 secs and 72 °C for 45 secs. The ∆∆CT method [47] was used to calculate the gene expression changes of the mixture- or PL-extract treated cells compared with untreated cells, and graphs were generated using GraphPad Prism, version 8 (GraphPad).

**Table 1:** Oligonucleotide sequences for qPCR analyses

Oligonucleotides	Sequences (5’-3’)
*MKI67* FW *MKI67* RW	CCACACTGTGTCGTCGTTTG CCGTGCGCTCATCCATTC
*MDM2* FW *MDM2* RW	GGACTCAGGTACATCTGTGAGTG CCTGTCTCACTAATTGCTCTCCTTC
*BRCA1* FW *BRCA1* RW	AAGAGGAACGGGCTTGGAA CACACCCAGATGCTGCTTCA
*HER2* FW *HER2* RW	ACCTGGTGGATGCTGAGG CACCATCAAATACATCGGAGCC
*TNFα* FW *TNFα* RW	CATCTTCTCGAACCCCGAGTG CTCGGCAAAGTCGAGATAGTCG
*EGFR* FW *EGFR* RW	AGACTCACTCTCCATAAATGCTACG CCAGTTCCTGTGGATCCAGAG
*PI3KCA* FW *PI3KCA* RW	CATCATGGTGAAAGACGATGGAC GCATTCTTGGGCTCCTTTACT
*KRAS* FW *KRAS* RW	GGATATTCTCGACACAGCAGGTC GCTAAGTCCTGAGCCTGTTTTGTG
*BAX* FW *BAX* RW	AGGATGCGTCCACCAAGAAG ACATGTCAGCTGCCACTCG
*CASP8* FW *CASP8* RW	CATAGAGATGGAGAAGAGGGTCATC ACTTCCTTCAAGGCTGCTGC
*GAPDH* FW *GAPDH* RW	GTCTCCTCTGACTTCAACAGCGA CCTGTTCGTGTAGCCAAATTCGT

### Statistical analysis

PSS, PL, and Cisplatin treated-4 breast cancer cell lines, including untreated cells, were tested in 5 independent replicates for cytotoxicity, IC50 determination, and BrdU assay. Whereas 4 independent replicates were undertaken for the colony formation assay, cell cycle analysis using PI, Annexin V apoptotic assay, and gene expression analysis. For statistical analysis, unpaired sample t-tests were applied to calculate the 95% CIs compared between each treatment group and untreated group.

## Results

### Characterization of extracts

To obtain chemical components derived from PSS and PL and to increase the contact of active components to the BC cell surface, dry PSS and PL powder were extracted using boiling water as a solvent in a Soxhlet extractor. Standard graphs using anthrone and Folin-Ciocalteu’s phenol reagents were generated to calculate the polysaccharide and phenolic content (Supplementary Fig. 1). The extraction yield of PL was 0.87 g/g dry weight of raw material which was 9-time higher than that of the PSS extract, 0.09 g/g. Almost all PL extract was polysaccharide (92.34 ± 7.15 g/ 100 g dry weight of PL extract), with only a trace of phenolic compounds (1.07 ± 0.02 g GAE/ 100 g dry weight of PL extract). In contrast, the total phenolic content of PSS extract was 11-time higher (11.91 0.20 g GAE/100 g), while the polysaccharide content (61.11 6.75 g/g) was 1.5-time lower than that of PL extract. The LC/QTOF-MS chromatogram of the PL extract also exhibited a lower intensity than that of PSS extract as shown in Supplementary Fig. 2. The metabolites in crude extracts of PSS and PL were tentatively identified using an untargeted screening method and the components with identification scores more than 80% are summarized in Table 2.

**Table 2:** The proposed compounds from PSS and PL extracts corresponding to the LC/QTOF-MS chromatographic peaks

No	Proposed compounds	Formula	Retention time (min)	Matching Score (%)	MS(n) Isol. m/z
***PSS extract***					
1	Benzaldehyde	C_7_H_6_O	13.23	80.1	107.0500
2	Asarone	C_12_H_16_O_3_	15.18	81.2	200.2389
	Ethyl caffeate	C_11_H_12_O_4_		80.7	
3	Sparteine	C_15_H_26_N_2_	16.62	80.7	235.1716
4	Lupanine	C_15_H_24_N_2_O	16.63	88.1	249.1873
	Matrine	C_15_H_24_N_2_O		83.4	
5	Formononetin	C_16_H_12_O_4_	16.81	80.2	269.2111
6	Tectorigenin	C_16_H_12_O_6_	17.37	80.2	301.1428
7	Rhoifolin	C_27_H_30_O_14_	17.38	81.3	579.2983
8	Citrusin	C_16_H_22_O_7_	17.68	80.7	327.2535
9*	(9Z)-9-Octadecenoic acid	C_18_H3_4_O_2_	17.77	80.9	283.2260
10*	5-Methoxyindole-3-acetic acid	C_11_H_11_NO_3_	17.79	81.7	228.2332
11*	Angelol A	C_20_H_24_O_7_	18.44	81.3	399.3114
	7-Ethylcamptothecin	C_22_H_20_N_2_O_4_		80.9	
12	4,5-Di-O-caffeoylquinic acid	C_25_H_24_O_12_	18.81	80.2	517.3758
13	Methyl 2-((2-methyl-4-oxo-3-phenoxy-4H-chromen-7-yl)oxy)acetate	C_19_H_16_O_6_	18.94	81.5	341.2698
14	Oleoyl Oxazolopyridine	C_24_H_36_N_2_O_2_	19.61	88.1	385.2961
15	Wortmannin	C_23_H_24_O_8_	19.62	86.3	429.3233
16	27-Hydroxycholesterol	C_27_H_46_O_2_	19.79	93.2	425.2900
17	Iridin	C_24_H_26_O_13_	20.21	82.0	545.4056
18	Rosmanol	C_20_H_26_O_5_	20.23	81.2	369.2999
19	Stearamide	C_18_H_37_NO	21.35	94.4	284.267
20	Metolachlor	C_15_H_22_ClNO_2_	21.49	80.0	310.3124
21*	Brevicarine	C_19_H_23_N_3_O	21.53	84.1	310.3130
22	13Z-Docosenamide	C_22_H_43_NO	22.15	86.4	338.3438
***PL extract***					
1	Benzaldehyde	C_7_H_6_O	13.29	80.0	107.0493
2	Asarone	C_12_H_16_O_3_	15.21	80.6	209.1545
	Ethyl caffeate	C_11_H_12_O_4_		80.0	
3	Sparteine	C_15_H_26_N_2_	16.64	80.6	235.1705
4	Lupanine	C_15_H_24_N_2_O	16.65	85.4	249.1862
	Matrine	C_15_H_24_N_2_O		83.4	
5	Formononetin	C_16_H_12_O_4_	16.84	80.2	269.2109
6	Tectorigenin	C_16_H_12_O_6_	17.38	80.9	301.1428
7	Rhoifolin	C_27_H_30_O_14_	17.39	81.3	579.2966
8	Citrusin	C_16_H_22_O_7_	17.66	80.5	327.2530
9	4,5-Di-O-caffeoylquinic acid	C_25_H_24_O_12_	18.85	80.0	517.3737
10	Methyl 2-((2-methyl-4-oxo-3-phenoxy-4H-chromen-7-yl)oxy)acetate	C_19_H_16_O_6_	18.97	81.5	341.2681
11	Oleoyl Oxazolopyridine	C_24_H_36_N_2_O_2_	19.63	87.7	385.2948
12	Wortmannin	C_23_H_24_O_8_	19.65	80.0	429.3211
13	27-Hydroxycholesterol	C_27_H_46_O_2_	19.74	93.2	425.2900
14	Iridin	C_24_H_26_O_13_	20.21	82.0	545.4056
15	Rosmanol	C_20_H_26_O_5_	20.25	81.2	369.2999
16	Stearamide	C_18_H_37_NO	21.35	94.4	284.267
17	Metolachlor	C_15_H_22_ClNO_2_	21.49	80.0	310.3124
18	13Z-Docosenamide	C_22_H_43_NO	22.12	86.4	338.3438

### Determination of the cytotoxicity of PSS extract, PL extract, and cisplatin on BC cell lines

**Fig. 1:**
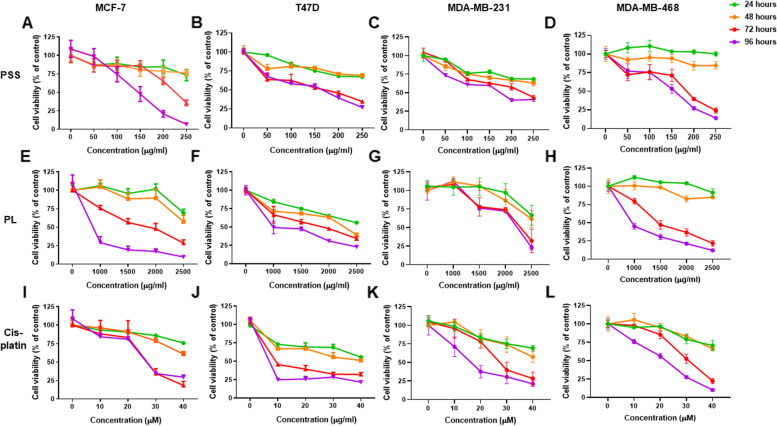
The cytotoxic of PSS and PL exposure in dose- and time-dependent on 4 breast cancer cell lines by MTT assay. Cisplatin was used as a positive control. **A**, **E**, and **I** MCF7, **B**, **F**, and **J**) T47D, **C**, **G**, and **K** MDA-MB-231, and **D**, **H**, and **L** MDA-MB-468 cell line. The trend observation showed in line graph where treatment for 24, 48, 72 and 96 h were depicted in green, orange, red, and purple, respectively. Error bar denoted as SE derived from 5 replicates for each dose

PSS and PL extracts were then used to treat MCF7, T47D, MDA-MB-231, and MDA-MB-468 BC cell lines in a dose- and time- dependent manner to determine the optimal conditions to be used for all subsequent experiments. The BC cell lines were exposed to PSS (0–250 µg/ml) and PL (0–2,500 µg/ml) extracts for 24, 48, 72, and 96 h, respectively, and MTT assay was then performed to observe the cells. Cisplatin (0–40 µM) was used as a positive control, as it has been shown to affect BC cell proliferation and apoptosis [48]. The results revealed that, after 72 h, the cells were significantly less viable following PSS, PL, and cisplatin treatment, as they had grown < 50% compared with the untreated cells (Fig. 1 and Supplementary Fig. 3 to 5). Therefore, the half-maximal inhibitory concentration (IC_50_) values for each of the treatment groups were investigated to identify the cytotoxicities of the extracts at 72 h post-treatment. Cell morphology was observed underneath the microscope; however, we did not find the substantial change in cell morphology of all breast cancer cell lines (Supplementary Fig. 6).

**Fig. 2:**
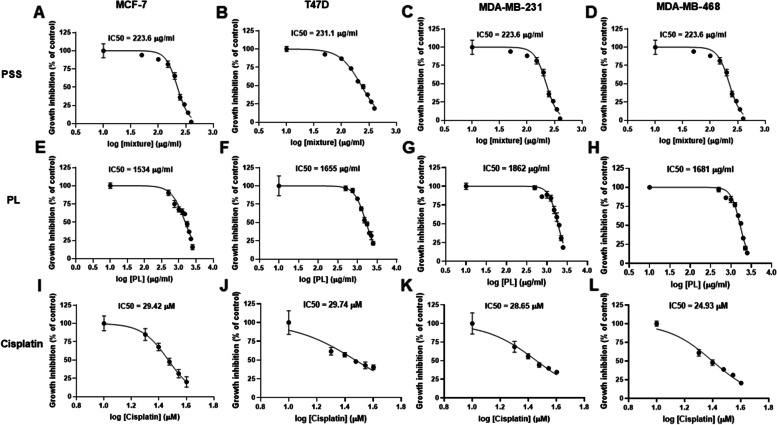
Half maximal inhibitory concentration (IC_50_) of PSS and PL determination on 4 breast cancer cell lines, 72-h post-exposure by MTT assay. Cisplatin was used as a positive control. Cell viability was calculated and reported as dose-inhibitory curve. IC_50_ was generated by GraphPad Prism 8 software. **A**, **E**, and **I** MCF7, **B**, **F**, and **J** T47D, **C**, **G**, and **K** MDA-MB-231, and **D**, **H**, and **L** MDA-MB-468 cell line. The experiment was performed for 5 independent replicates, data represented as mean ± SE

The IC_50_ values identified in the experiments where the MCF7, T47D, MDA-MB-231 and MDA-MB-468 cell lines were treated with PSS, PL, and cisplatin for up to 72 h are shown in the dose-inhibitory curves in Fig. 2, and these data are summarized in Table 3; moreover, the 95% CIs are also presented. Subsequently, the exact concentrations used in experiments were close to the observed IC_50_ values and fell within the 95% CI ranges. Specifically, the working concentrations of the extracts used for experiments with the MCF7, T47D, MDA-MB-231 and MDA-MB-468 cell lines were 225, 230, 250 and 160 µg/ml for PSS, 1,550, 1,650, 1,850 and 1,700 µg/ml for PL, and 30, 30, 30 and 25 µM for cisplatin, respectively.

**Table 3:** The cytotoxicity of PSS extract, PL extract, and cisplatin represented as IC_50_, including 95%CI on BC cell lines

**Breast cancer cell line**	**IC**_**50**_** values (95% CI)**		
	**PSS (µg/ml)**	**PL (µg/ml)**	**Cisplatin (µM)**
**MCF7**	223.6 (211.1 – 235.6)	1534 (1444 – 1626)	29.42 (27.41 – 31.50)
**T47D**	231.1 (222.7 – 239.6)	1655 (1559 – 1757)	29.74 (25.95 – 34.93)
**MDA-MB-231**	248.8 (234.1 – 263.7)	1862 (1790 – 1936)	28.65 (25.66 – 32.14)
**MDA-MB-468**	150.3 (133.7 – 166.7)	1681 (1631 – 1731)	24.83 (23.78 – 26.07)

### PSS and PL extracts possess anti-cell proliferative activity

**Fig. 3:**
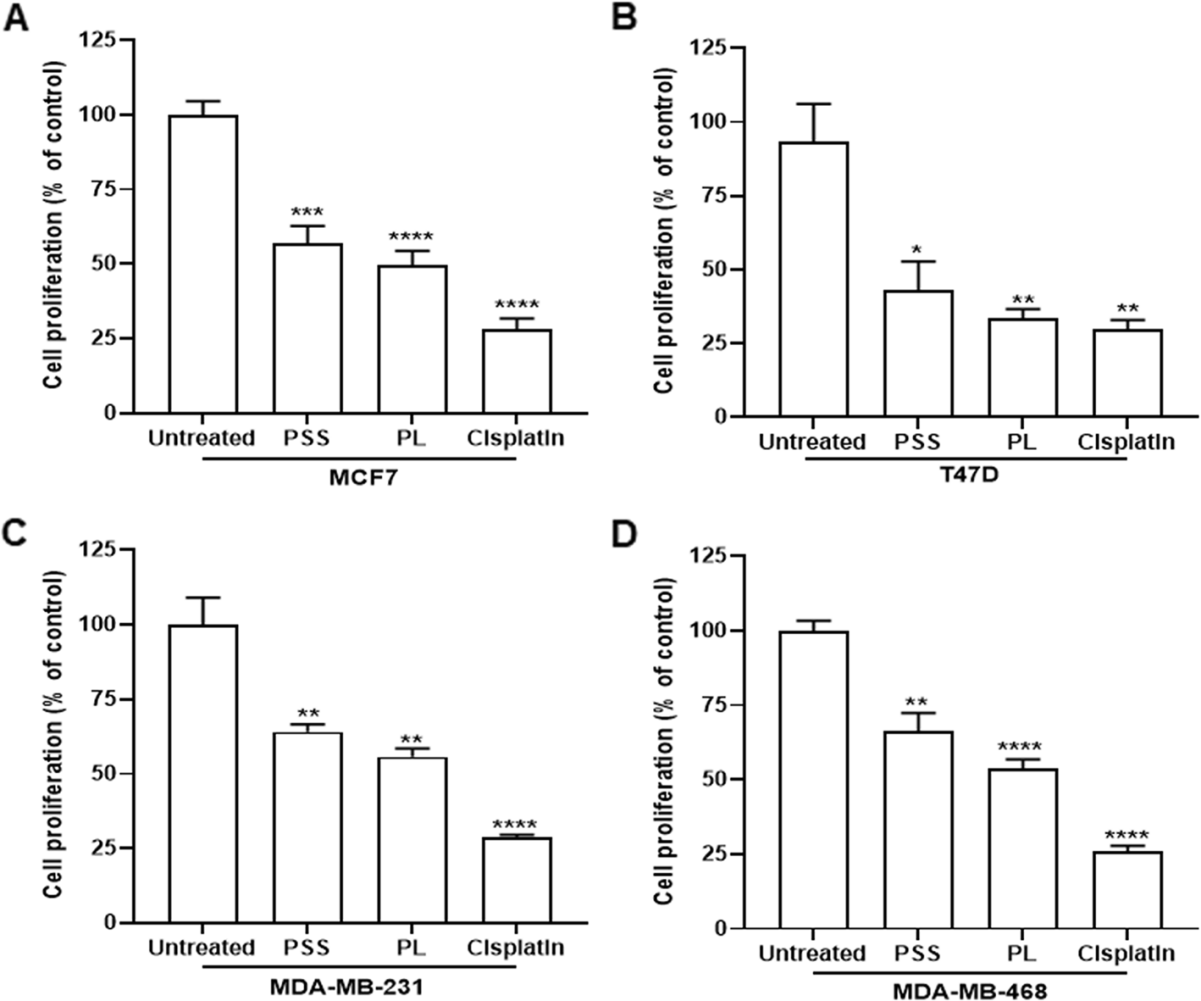
Anti-proliferative activity of PSS and PL on 4 breast cancer cell lines. Cisplatin was used as a positive control. BrdU colorimetric assay was tested 72-h post treatment, the percentage cell proliferation was plotted in % of control compared between the untreated cells and each treatment group. **A** MCF7, **B** T47D, **C** MDA-MB-231, and **D** MDA-MB-468. Data represented as mean ± SE (*n* = 5) where ^*^, ^**^, ^***^, ^****^ represented *P* ≤ 0.05, *P* ≤ 0.01, *P* ≤ 0.001, and *P* ≤ 0.0001 from statistical analysis using Unpaired t-test compared between the untreated cells among the same group

To investigate the effect of the extracts on BC cell proliferation, an ELISA BrdU colorimetric assay was performed, using the IC_50_ concentrations described above. The results are presented as the percentages of inhibition of cell proliferation in PSS-, PL- and cisplatin-treated cells compared with untreated cells. Exposure to PSS led to a significant decrease in the proliferation of the MCF7, T47D, MDA-MB-231 and MDA-MB-468 cell lines from 100% (untreated cells) to 56.87, 39.91, 64.03 and 66.45%, respectively. Regarding treatment with PL, significant decreases in the proliferation of the same cell lines were also observed to 49.60, 37.03, 55.69 and 53.84%, respectively (Fig. 3A-D).

**Fig. 4:**
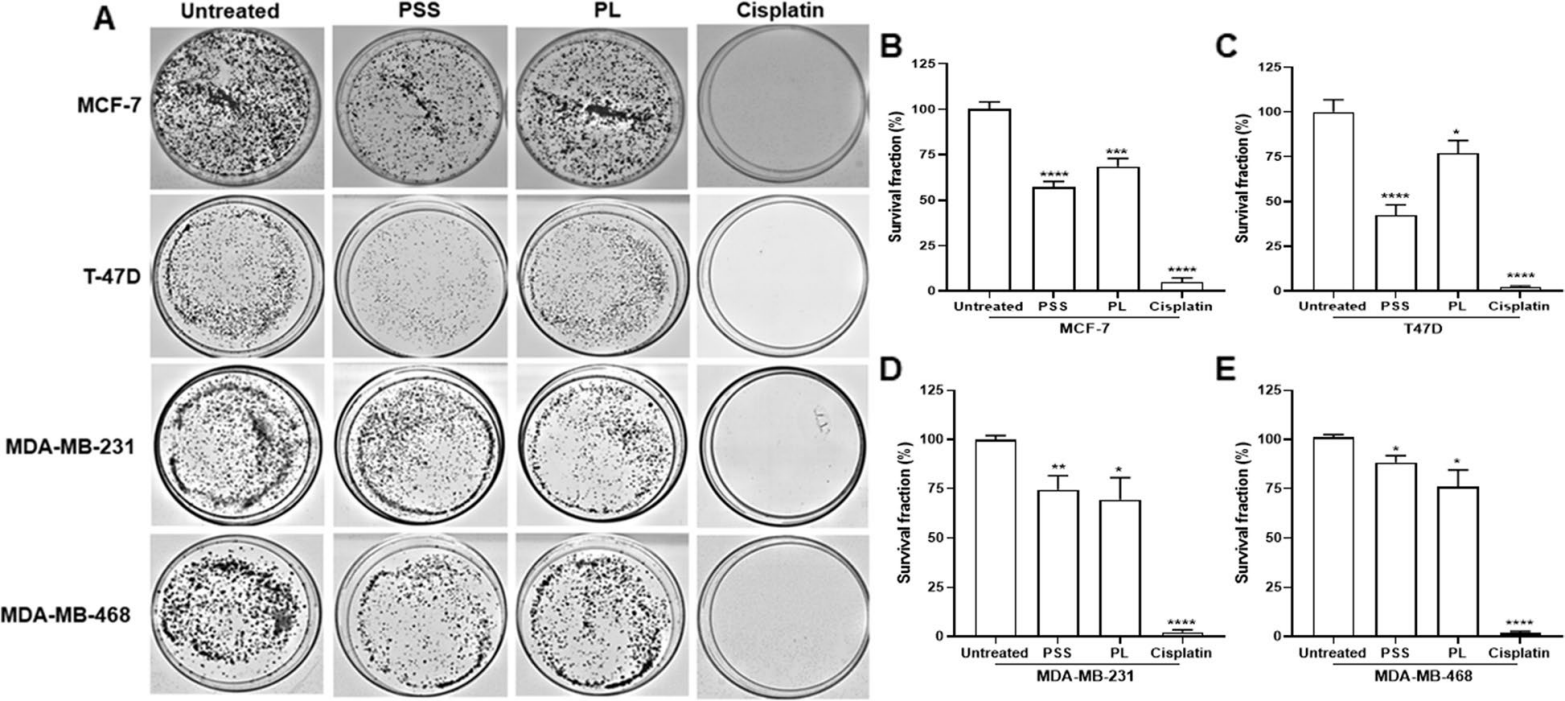
A long-term inhibitory effect of PSS and PL treatments on 4 breast cancer cell lines tested by colony formation assay. Cisplatin was used as a positive control. **A** After 72-h treatment and further cell cultivation for 21 days, BC colonies were formed. The percentage of survival cells were calculated and plotted as shown in graph **B** MCF7, **C** T47D, **D** MDA-MB-231, and **E** MDA-MB-468 cell line. Data represented as mean ± SE; ^*^, ^**^, ^***^, ^****^ represented *P* ≤ 0.05, *P* ≤ 0.01, *P* ≤ 0.001, and *P* ≤ 0.0001 from statistical analysis using Unpaired t-test compared between the untreated cells among the same group

Moreover, to address the potential long-term inhibitory effects of PSS and PL on exposure to the BC cell lines, a colony formation assay was performed. After treatment for 72 h, cells were collected, seeded into a 60 mm dish at a density of 1 × 10^3^ cells/ml, further cultured for 14 days, stained with crystal violet, and then colonies were counted. The results obtained indicated that the colony numbers of all BC cell lines were significantly decreased following treatment with PSS and PL, also including the cisplatin treatment group, compared with untreated cells (Fig. 4). PSS administration reduced the relative MCF7, T47D, MDA-MB-231 and MDA-MB-468 colony number percentages to 57.23, 42.29, 75.51 and 88.24%, respectively (cf. 100% for the untreated cells). Similar results were obtained upon PL treatment: the colony number percentages of the four BC cell lines investigated decreased to 68.58, 76.10, 69.41, and 77.04%, respectively. Following cisplatin treatment, the BC colonies were strongly compromised as the colony number percentages were close to 0% (Fig. 4B-E). It was noteworthy that, in terms of short-term observations, PL administration elicited lower BC cell proliferation rates compared with PSS; however, in a long-term culture, the colony numbers of MCF7 and T47D were lower following PSS treatment compared with PL treatment. On the other hand, the short- and long-term inhibitory effects were relatively the same for the MDA-MB-231 and MDA-MB-468 cell lines.

### Effect of PSS and PL exposure on cell cycle arrest

**Fig. 5:**
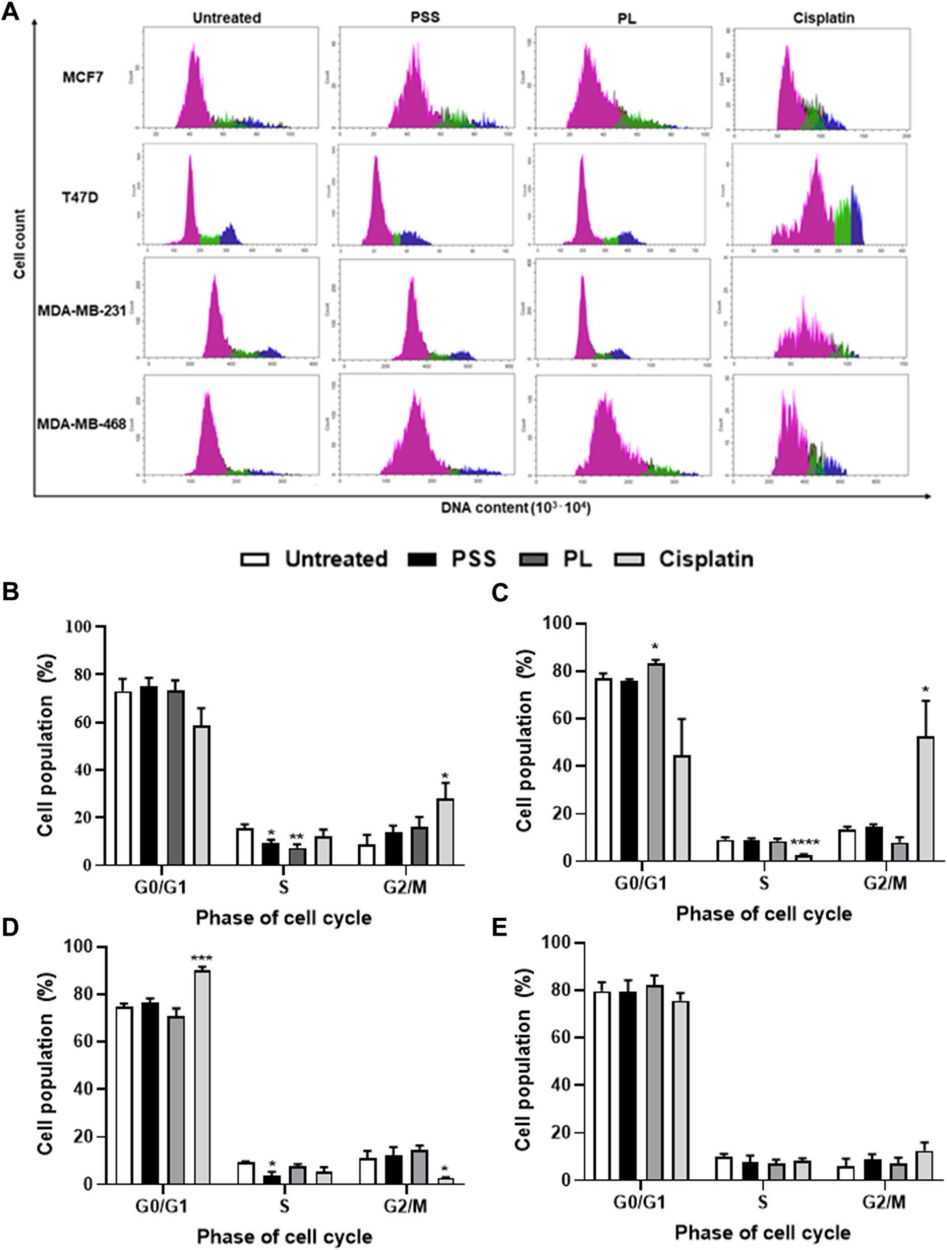
Effect of PSS and PL treatment for 72 h of 4 breast cancer cell lines on cell cycle. Cisplatin was used as a positive control. Cells were treated with RNase A and stained with propidium iodide (PI). **A** Histograms are representative of 4 biological replicates of DNA content calculating from PI staining versus BC cell number (count) from flow cytometry. The G0/G1 phase was pink S phase was green, and G2/M phase was blue. Graphs shown percentage of cell population in each cell cycle phase were drawn **B** MCF7, **C** T47D, **D** MDA-MB-231, and **E** MDA-MB-468. The results are provided as mean ± SE; ^*^, ^**^, ^***^, ^****^ represented *P* ≤ 0.05, *P* ≤ 0.01, *P* ≤ 0.001, and *P* ≤ 0.0001 from statistical analysis using Unpaired t-test compared between the untreated cells among the same group

To explore the association of PSS and PL with the BC cell cycle upon treatment of the cells with the IC_50_ concentrations of the extracts, PI staining and flow cytometric analysis were performed to measure the DNA content of each single cell, as shown in the histograms showing plots of the cell count vs. DNA content. The data obtained showed that treatment with both PSS and PL led to a significant reduction in the percentages of the S-phase cell population to 9.13 and 7.27%, respectively, from 15.68% of untreated MCF7 cells. Moreover, PSS and PL administration led to an increase in the cell populations occupying the G0/G1 and G2/M phases (Fig. 5A and B, and Table 4). However, with the T47D cell line, only treatment with PL caused a significant increase in the G0/G1 cell population from 77.15 to 83.14% (Fig. 5A and C and Table 4). Moreover, exposure to PSS induced a significant decrease in the MDA-MB-231 cell population only in S-phase (from 9.34 to 3.73%), with a concomitant trend of incrementally increasing the G0/G1 and G2/M cell populations (Fig. 5A and D and Table 4). Nevertheless, no significant effects of PSS and PL exposure on MDA-MB-468 cell line were observed. Regarding the MDA-MB-468 cell line, treatment with PSS tended to induce G2/M arrest, whereas treatment with PL appeared to influence G0/G1 arrest (Fig. 5A and E, and Table 4).

**Table 4:** The percentage of cell population in each phase of cell cycle resulted from PSS, PL, and cisplatin treatment in 4 breast cancer cell lines

**Breast cancer cell line**	**Cell cycle phase (Mean (%) ± SE)**		
	**G0/G1**	**S**	**G2/M**
**MCF7**			
** - Untreated**	73.02 ± 5.30	15.68 ± 1.53	8.90 ± 3.86
** - PSS**	74.94 ± 3.65	9.13 ± 1.55 ^*^	13.94 ± 2.61
** - PL**	73.27 ± 4.32	7.27 ± 1.50 ^**^	16.32 ± 3.97
** - Cisplatin**	58.56 ± 7.39	12.00 ± 2.93	28.06 ± 6.34 ^*^
**T47D**			
** - Untreated**	77.15 ± 2.00	9.07 ± 1.07	13.24 ± 1.34
** - PSS**	75.99 ± 0.68	8.76 ± 1.05	14.64 ± 0.94
** - PL**	83.14 ± 1.60 ^*^	8.55 ± 0.98	7.98 ± 2.16
** - Cisplatin**	44.47 ± 15.48	2.57 ± 0.43 ^****^	52.54 ± 15.06 ^*^
**MDA-MB-231**			
** - Untreated**	74.91 ± 1.22	9.34 ± 0.33	11.08 ± 3.02
** - PSS**	76.29 ± 1.92	3.73 ± 1.58 ^*^	12.07 ± 3.63
** - PL**	70.87 ± 3.19	7.83 ± 0.77	14.48 ± 1.90
** - Cisplatin**	90.12 ± 1.39 ^***^	5.43 ± 1.97	2.82 ± 0.25 ^*^
**MDA-MB-468**			
** - Untreated**	80.64 ± 2.70	8.19 ± 1.23	9.95 ± 1.54
** - PSS**	79.83 ± 4.90	7.38 ± 2.81	11.16 ± 1.62
** - PL**	78.33 ± 5.28	8.63 ± 1.93	8.67 ± 2.74
** - Cisplatin**	79.62 ± 4.30	8.03 ± 1.37	9.25 ± 1.75

^*^, ^**^, ^***^, ^****^ represented *P* ≤ 0.05, *P* ≤ 0.01, *P* ≤ 0.001, and *P* ≤ 0.0001 from statistical analysis using Unpaired t-test

### PSS and PL induce apoptosis

**Fig. 6:**
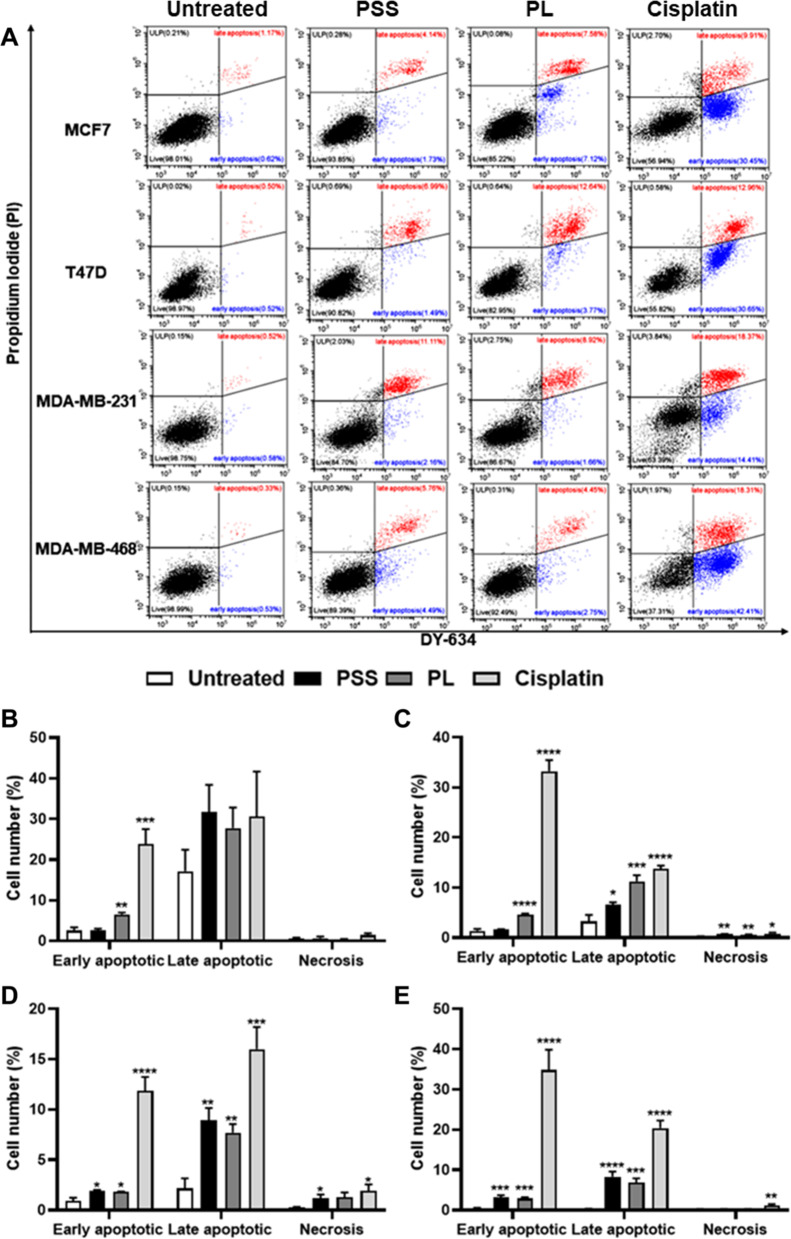
Effect of PSS and PL exposure of 4 breast cancer cell lines on apoptosis and necrosis. Cisplatin was used as a positive control. **A** Histograms shown are representative of 4 biological replicates of the untreated cells and 72-h treatment of PSS, PL, and Cisplatin was performed in 4 breast cancer cell lines. Cells were co-stained with the Annexin V-DY-634 and propidium Iodide (PI), the single cell population in each quadrant was generated. Graphs shown percentage of apoptotic and necrotic cells from each treatment were also plotted **B** MCF7, **C** T47D, **D** MDA-MB-231, and **E** MDA-MB-468. The results are provided as mean ± SE; ^*^, ^**^, ^***^, ^****^ represented *P* ≤ 0.05, *P* ≤ 0.01, *P* ≤ 0.001, and *P* ≤ 0.0001 from statistical analysis using Unpaired t-test compared between the untreated cells among the same group

To evaluate whether PSS and PL, which were shown to inhibit cell proliferation, were associated with the induction of apoptotic cell death, double staining of annexin V-conjugated DY 634 (AnV) and PI was executed. Images obtained from flow cytometric analysis can be divided into 4 quadrants according to ability of AnV to bind to phosphotidylserine residues on the inner cytoplasmic membrane; the AnV molecules are released to the exterior on account of membrane leakage in early apoptosis [49], and PI, which is able to bind to DNA, is detected following rupture of the cell membrane via PI staining of the DNA, which is represented in late apoptotic or necrotic cells. Therefore, in quadrant 1 (Q.1), AnV^+^/PI^+^, represented late apoptotic cells, AnV^−^/PI^+^ (Q.2) represented necrotic cells, AnV^−^/PI^−^ (Q.3) represented viable cells, and AnV^+^/PI^−^ (Q.4) represented early apoptotic cells. Post-treatment at 72 h, the data revealed that MCF7 and T47D early apoptotic cells were not induced by exposure to PSS; moreover, the extent of late apoptosis was only significantly increased in T47D cells, and not in MCF7 cells (Fig. 6A-C, and Table 5). However, only in the MDA-MB-231 and MDA-MB-468 cell lines did PSS cause a significant increase in the population of early apoptotic cells, followed by late apoptosis: specifically, from 0.93% of untreated cells to 1.87% cells in early apoptosis, and from 2.16% of untreated cells to 8.95% cells in late apoptosis, for the MDA-MB-231 cell line, and from 0.56% of untreated cells to 3.17% cells in early apoptosis, and from 0.28% of untreated cells to 8.33% cells in late apoptosis, for the in MDA-MB-468 cells (Fig. 6A, D and E, and Table 5). In terms of PL treatment, the results revealed markedly increased numbers of cells in early and late apoptosis for the T47D, MDA-MB-231 and MDA-MB-468 cell lines. Even though exposure to PL led to a significantly enhanced population of early apoptotic cells with the MCF7 cell line, this provided a trend of late apoptosis in MCF7 cell population without any significance (Fig. 6A and B, and Table 5). Aside from apoptosis, it was not possible to exclude the presence of necrotic cells: they were significantly induced by 0.71% after treatment with PSS, and by 0.65% following treatment with PL, in the T47D cell line, and by 1.19% following exposure to PSS in the MDA-MB-231 cell line (Fig. 6A, C and D, and Table 5). Treatment with cisplatin, used as a positive control, revealed an extensive reduction in the numbers of living cells contributing to early apoptosis in all four BC cell lines. However, cisplatin only affected late apoptosis significantly in the MCF7 cell line. Regarding the prospect of cisplatin toxicity in inducing necrotic cells, this was found to occur significantly only in the T47D and MDA-MB-231 cell lines (Fig. 6A-E, and Table 5).

**Table 5:** Cell population content (%) found in early apoptosis, late apoptosis, necrosis, and live cells caused by PSS, PL, and cisplatin exposure 4 breast cancer cell lines

**Breast cancer cell line**	**Cell population (Mean (%) ± SE)**			
	**Viable cells**	**Early Apoptotic cells**	**Late Apoptotic cells**	**Necrotic cells**
**MCF7**				
** - Untreated**	87.74 ± 5.12	2.53 ± 0.81	22.41 ± 0.80	0.57 ± 0.22
** - PSS**	78.72 ± 7.34	2.55 ± 0.48	31.66 ± 6.74	0.70 ± 0.35
** - PL**	75.59 ± 4.70	6.52 ± 0.47 ^**^	27.77 ± 5.05	0.32 ± 0.15
** - Cisplatin**	54.78 ± 4.65 ^***^	23.76 ± 3.76 ^***^	30.56 ± 11.09	1.41 ± 0.44
**T47D**				
** - Untreated**	96.06 ± 1.81	1.39 ± 0.40	3.24 ± 1.30	0.24 ± 0.09
** - PSS**	91.06 ± 0.62 ^*^	1.67 ± 0.09	6.56 ± 0.52 ^*^	0.71 ± 0.07 ^**^
** - PL**	83.57 ± 1.38 ^***^	4.55 ± 0.28 ^****^	11.24 ± 1.24 ^***^	0.65 ± 0.04 ^**^
** - Cisplatin**	52.18 ± 2.88 ^****^	33.26 ± 2.20 ^****^	13.73 ± 0.70 ^****^	0.82 ± 0.21 ^*^
**MDA-MB-231**				
** - Untreated**	96.65 ± 1.34	0.93 ± 0.29	2.16 ± 0.99	0.26 ± 0.07
** - PSS**	87.99 ± 1.63 ^**^	1.87 ± 0.13 ^*^	8.95 ± 1.19 ^**^	1.19 ± 0.37 ^*^
** - PL**	89.21 ± 1.20 ^**^	1.80 ± 0.05 ^*^	7.70 ± 0.86 ^**^	1.30 ± 0.47
** - Cisplatin**	70.22 ± 3.49 ^****^	11.87 ± 1.38 ^****^	15.99 ± 2.23 ^***^	1.92 ± 0.64 ^*^
**MDA-MB-468**				
** - Untreated**	99.08 ± 0.11	0.56 ± 0.03	0.28 ± 0.07	0.28 ± 0.19
** - PSS**	88.01 ± 1.41 ^****^	3.17 ± 0.54 ^***^	8.33 ± 1.23 ^****^	0.24 ± 0.05
** - PL**	90.11 ± 1.26 ^****^	2.90 ± 0.32 ^****^	6.80 ± 1.12 ^***^	0.23 ± 0.04
** - Cisplatin**	43.64 ± 5.69 ^****^	34.81 ± 5.04 ^****^	20.40 ± 1.89 ^****^	1.19 ± 0.28

^*^, ^**^, ^***^, ^****^ represented *P* ≤ 0.05, *P* ≤ 0.01, *P* ≤ 0.001, and *P* ≤ 0.0001 from statistical analysis using Unpaired t-test

### Effect of PSS and PL on BC marker genes, genes involved in cell proliferation and the cell death pathway

**Fig. 7:**
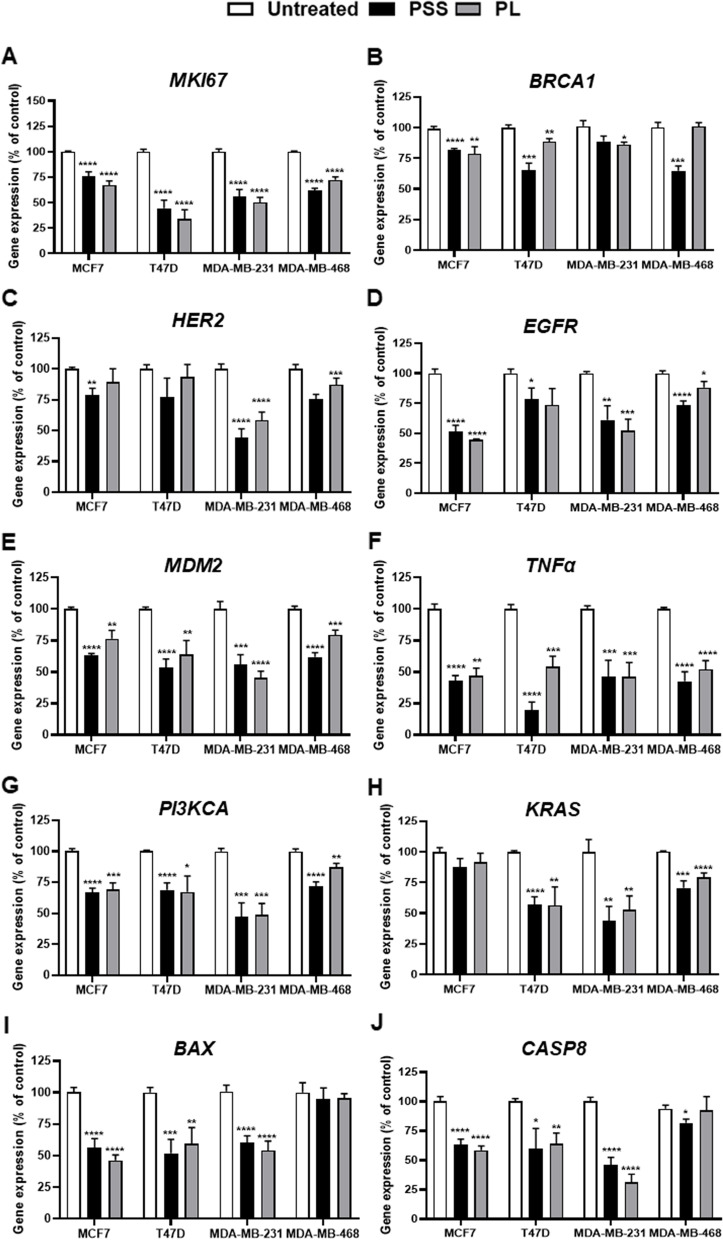
The expression changes of BC biomarker gene, gene-involved in cell proliferation and apoptosis in mRNA level in 4 breast cancer cell lines after PSS and PL treatment. **A** *MKI67,* **B** *BRCA1*, **C** *HER2*, **D** *EGFR*, **E** *MDM2*, **F** *TNFα*, **G** *PI3KCA*, **H** *KRAS*, **I** *BAX*, and **J** *CASP8*. Data represents 4 independent replicates as mean ± SE; ^*^, ^**^, ^***^, ^****^ represented *P* ≤ 0.05, *P* ≤ 0.01, *P* ≤ 0.001, and *P* ≤ 0.0001 from statistical analysis using Unpaired t-test compared between the untreated cells among the same group

To gain insights into which underlying mechanisms may be responsible for mediating the effects elicited by PSS and PL, an exploration of the mRNA expression of highly penetrant genes, genes with high mutation rates and tumor markers in breast carcinogenesis was conducted by RT-qPCR following PSS and PL treatment for 72 h. *MKI67* was reduced in PSS and PL-treated BC cell lines. For PSS-treated cells, the expression level of *MKI67* was reduced to 75.12, 44.38, 55.96 and 61.56% (*P* ≤ 0.0001 in all BC cell lines) while PL-treated cells lead to the reduction of MKI67 expression of 69.24, 33.89, 50.29 and 72.26% (*P* ≤ 0.0001 in all BC cell lines) in MCF-7, T47D, MDA-MB-231 and MDA-MB-468, respectively (Fig. 7A). Moreover, the data obtained demonstrated that PSS reduced the level of *BRCA1* expression in MCF7 cells (*P* ≤ 0.0001), T47D cells (*P* = 0.0003) and MDA-MB-468 cells (*P* = 0.0143). It appeared that treatment with PL elicited a less significant (although still significant at the level *P* ≤ 0.05) reduction in the expression level of *BRCA1* in MCF7 cell compared with PSS (*P* = 0.0081); similar trends were observed with T47D (*P* = 0.0059) and MDA-MB-231 (*P* = 0.00145) cells. No significant differences were observed in PL-treated MDA-MB-468 cells (Fig. 7B).

Upon PSS treatment, the expression of *HER2* was significantly reduced in MCF7 (*P* = 0.0023) and MDA-MB-231 (*P* ≤ 0.0001) cells; decreases were also apparent with T47D and MDA-MB-468 cells (Fig. 7C). The expression level of *EGFR* was found to be dramatically reduced in PSS- and PL-treated MCF7 cells (*P* ≤ 0.0001 for both treatments), MDA-MB-231 cells (*P* = 0.007 and *P* = 0.0004 for PSS and PL, respectively) and MDA-MB-468 cells (*P* ≤ 0.0001 and *P* = 0.0404, respectively). However, a significant decrease in expression of *EGFR* was only found in PSS-treated T47D cells (*P* = 0.0467) (Fig. 7D).

According to our results, exposure of the four tested BC cell lines to PSS and PL led to marked decreases in *MDM2* expression. Significant decreases were elicited in MCF7 cells (*P* ≤ 0.0001 and *P* = 0.0017 for the PSS and PL treatments, respectively), T47D cells (*P* ≤ 0.0001 and *P* = 0.0053), MDA-MB-231 cells (*P* = 0.0002 and *P* ≤ 0.0001) and MDA-MB-468 (*P* ≤ 0.0001 and *P* = 0.0002) (Fig. 7E). However, a significant level of upregulation of *TP53* was only observed in PL-treated MCF7 cells, although an upward trend was also found in PSS-treated T47D cells (Supplementary Fig. 7). A reduction in *TNFα* expression following exposure to PSS and PL was also identified in MCF7 cells (*P* ≤ 0.0001 and *P* = 0.0011 for PSS and PL treatments, respectively), T47D cells (*P* ≤ 0.0001 and *P* = 0.0005), MDA-MB-231 cells (*P* = 0.0007 and *P* = 0.0002) and MDA-MB-468 cells (*P* ≤ 0.0001 and *P* ≤ 0.0001) (Fig. 7F).

Moreover, our data suggested that PSS and PL significantly contributed to a reduction in *PI3KCA* expression in MCF7 cells (P ≤ 0.0001 and P = 0.0001 for PSS and PL, respectively), T47D cells (*P* ≤ 0.0001 and *P* = 0.0226), MDA-MB-231 cells (*P* = 0.0005 and *P* = 0.0002) and MDA-MB-468 cells (*P* ≤ 0.0001 and *P* = 0.0037) (Fig. 7G). Treatment with PSS led to lower expression level of *PI3KCA* and *KRAS* compared with PL treatment in the three BC cell lines, except T47D. *KRAS* expression was significantly downregulated after treatment in T47D (*P* ≤ 0.0001 and *P* = 0.0034 for PSS and PL treatments, respectively), MDA-MB-231 cells (*P* = 0.0036 and *P* = 0.0096) and MDA-MB-468 (*P* = 0.0002 and *P* ≤ 0.0001); however, only a slightly decrease in *KRAS* expression was observed with PSS- and PL-treated MCF7 cells (Fig. 7H).

Owing to the discovery that PSS and PL could induce cell apoptosis, genes involved in intrinsic and extrinsic apoptosis, *BAX* and *CASP8* expression levels were investigated. However, these experiments failed to identify any upregulation of either *BAX* or *CASP8* expression in the four PSS- and PL-exposed BC cell lines (Fig. 7I and J).

## Discussion

The present study has investigated the capabilities of a PSS mixture in comparison to PL on BC treatment. PL, SC, SG possess anti-cancer activities due mainly to the existence of various active ingredients. SC and SG contained phenolic compounds contributing to the prominent properties on anti-oxidation and anti-inflammation [50]. Durgo et al. [51] discovered that antioxidant activity was strongly connected with the phenolic content of mushroom blend, and that the higher level of the antioxidant generated, the greater cytotoxicity [52]. However, PL's predominant active component is polysaccharide, especially β-glucan, which primarily affects anti-proliferation and modulates immune response [52]. These interconnected findings lead us to speculate if the combination of SC and SG as a source of phenolic compound and PL as a source of polysaccharides (a mixture denoted as PSS) will result in increased cytotoxicity and suppression of cell growth in breast cancer cells. Thus, we confirmed the phenotypic alteration and investigated the underlying molecular mechanism of four BC cell lines after PSS or PL treatments. The findings are discussed in better detail below.

The capabilities of a PSS mixture (PL + SC + SG; 3:1:1) with individual PL in BC treatment were investigated. To obtain the polysaccharide, PSS and PL powders were extracted with hot water, and the tentative compounds in the extracts were analyzed using LC/QTOF-MS. A total of 18 and 22 peaks were identified from the chromatograms of PL and PSS extracts, respectively, with 8 unknown peaks. Among these, a glycoside flavonoid, iridin (RT at 20.21 min), that has been reported to have antioxidant, anti-inflammatory, and anti-cancer properties was identified as a major compound from both extracts [53]. The additional compounds in the PSS extract were identified as (9Z)-9-octadecenoic acid, 5-methoxyindole-3-acetic acid, angelol A, and brevicarine which have been reported as the major compounds from the ethanolic extracts of SC and SG [50]. Most of the tentative compounds in this study were recognized to have anti-cancer activities, including ethyl caffeate [54], sparteine [55], matrine [56], formononetin [57], rhoifolin [58], rosmanol, and angelol A [59], with different mechanisms.

Our experiments were performed in BC cell lines that were selected as follows: MCF7 and T47D are categorized as Progesterone receptor (PR) + /Estrogen receptor (ER) + /HER2 expression- cell lines, whereas the MDA-MB-231 and MDA-MB-468 cell lines are TNBC [60]. Overall, TNBC tends to show the worst survival rates with more aggressive phenotypes, and intermediate response to chemotherapy [61]. Therefore, in choosing these cell lines, it was feasible to observe if PSS and PL may influence BC with diverse genetic backgrounds.

A prior study demonstrated the action of PL extract in many human cancer cell lines, including MCF7. It showed that MCF7 growth rate was reduced by 40% after 72 h of 250 µg/ml PL treatment [62]. These findings were consistent with our study, which revealed that PL reduced the viability and proliferation of BC cell lines in a dose- and time-dependent manners (Fig. 1). In addition, the IC_50_ concentration of PL used in this study was between 1,400 and 1,600 µg/ml (Table 3), which was very similar to IC_50_ value of another study performed in MCF7 and MDA-MB-231 cells, which demonstrated that PL is able to suppress growth and angiogenesis [63]. Differences in the dose of action used for PL depend on which part of PL is extracted; i.e., the fruiting body or mycelium; the different parts of the fungus provide differing bioactive agent groups and contents [64], and the solvent used in herbal preparation also affects the dose/properties of PL [65]. For example, previous studies showed that PL extracted by ethanol exhibited strong antioxidant and anti-angiogenic properties [66], whereas aqueous PL extract impacted growth and angiogenesis inhibition [63]. PL in this study, obtained using distilled H_2_O as solvent, was found to inhibit all investigated BC cell lines (Figs. 1, 2 and 3).

Noticeably, compared with PL, the PSS was found to be effective with a lower dose of action (130–265 µg/ml), based on experiments performed in each cell line (Table 3). The highest dose of PSS was used for the MDA-MB-231 cell line (TNBC; claudin-low subtype), whereas MDA-MB-468 cell line (TNBC; basal-like subtype) responded to the lowest dose of PSS compared with the other BC cell lines. This might be due to differences in their molecular features. MDA-MB-468 cells possess high levels of Ki67 expression; however, this cell line is not as invasive as MDA-MB-231, which is enriched in epithelial-mesenchymal transition (EMT) [60, 67]. The luminal A subtype cell lines, MCF7 and T47D, responded to PSS treatment equally as well, with respect to the IC_50_ concentrations. Notably, PL treatment conferred more effective growth inhibition of the BC cell lines compared with PSS, as confirmed by BrdU assay (Fig. 3). This finding was in agreement with detection of *MKI67* expression (Fig. 7A), with the exception of the MDA-MD-468 cell, for which PL was observed to yield a higher level of *MKI67* expression relative to PSS (Fig. 7A). According to our results, both PSS and PL functioned well in terms of their anti-proliferative activities. PL contains a higher ß-glucan content compared with PSS. ß-glucan exerts its action mainly on the suppression of cell proliferation. Hence, it was expected that fine PL would show a prominent response in terms of the inhibition of cell proliferation, whereas PSS would influence functions other than anti-tumor growth, including having anti-inflammatory and antioxidant properties.

Moreover, to eliminate the possibility that PSS and PL were not functioning solely in a short-acting manner on cell proliferation, anchorage-independent growth was evaluated by colony formation assay after 20 days of treatment. PSS caused a marked reduction in the number of colonies of MCF7 and T47D cells. Even though PSS and PL led to a significant decrease in MDA-MB-231 and MDA-MB-468 cell proliferation tested by BrdU assay and detection of *MKI67* mRNA expression, treatment with PSS and PL did not significantly affect the colony numbers of these cell lines. Following chemotherapeutic cisplatin treatment, the colony numbers were reduced to almost 0% for all four BC cell lines (Fig. 4). Admittedly, cisplatin administration brings about adverse side effects to patients [68, 69]; in addition, long-term usage of cisplatin contributes to drug resistance [70, 71]. With the goal of improving the therapeutic regimen, the findings of the present study have consequently shed light on the potential adjuvant use of PSS or PL and chemotherapeutic agents for the remedy of BC. As previously reported [72], PL was also suggested for use as an adjuvant chemo-medication for treatment of pancreatic cancer after surgical resection.

Subsequently, differential effects on cell cycle arrest upon exposure to PSS and PL were identified in the present study, without each cell cycle phase being concomitantly affected. Almost all investigated cells tended to accumulate in G2/M after 72 h of PSS and PL treatment, except T47D which PL treatment significantly contributed to cell accumulation in G0/G1 (Fig. 5). Cell cycle is driven by many regulatory proteins, and the varied expression of their genes; for example, cyclins or CDK protein family members. Previously, the connection between Ki-67 and other cell cycle genes was studied using bioinformatics, including a direct correlation between cyclin A2 and Ki-67 expression at both the mRNA and protein levels was found in breast cancer cell line. The Ki-67 levels were low in quiescent cells and cells entering the cell cycle. In addition, the elevated Ki-67 expression was a late indicator of cell-cycle entry, with its highest levels occurring during the G2/M phases [73]. The Ki-67 isoform may differentially influence cell proliferation and cell cycle progression [74], the certain isoforms of Ki-67 can trigger the translocation of cyclin B from the cytoplasm to the nucleolus [75] in which cyclin B is the main component of G2/M transition of the cell cycle [76]. According to our results, both of PSS and PL-treated BC cell lines contributed to a decrease of *MKI67* expression (Fig. 7A) implying that our extracts have a potential to suppress breast cancer cell proliferation through cell cycle arrest.

Cell cycle arrest is coupled with apoptosis for the maintenance of tissue homeostasis [77]. Moreover, cells undergoing apoptosis are exploited in anti-cancer strategies [78]. Hence, PSS- and PL-induced apoptosis were investigated in the present study using co-staining of annexin V-DY-634/PI and flow cytometric analysis. The results obtained suggested that PL is able to significantly induce both early- and late apoptosis in all the four BC cell lines, findings that are in line with previous studies [63, 79]. However, PSS exposure resulted in accumulation of early and late apoptotic cells only in the TNBC cell lines. It is of interest that PSS gave rise to significant numbers of late apoptotic cells only with the T47D cell line (Fig. 6). One characteristic of early apoptosis is the exposure of phosphatidylserine on the outer layer of ruptured cell membranes. During the apoptotic process, changes in cell morphology, activation of caspases and chromatin condensation eliciting DNA fragmentation are processes that occur in the late apoptotic phase. Furthermore, neither early nor late apoptosis result in the triggering of inflammatory cellular components; rather, the inflammatory molecules are induced during necrosis, which may arise from the degradation of organelle leakage of the remnants of apoptotic cells [80, 81]. Several studies have reported that DNA fragmentation can be observed without any plasma membrane disruption, and certain drugs may disturb cellular effects, resulting in an imbalance of DNA damage and cell growth, in which cases apoptosis may not always be observed [82]. Of note, the percentage of apoptosis in all PSS and PL-treated cells was little changed (about 1–7%) compared to untreated cells. Consequently, the PSS and PL extracts would not have a prominent effect on apoptosis. In this study, to observe apoptotic pathway in molecular levels, *BAX* and *CASP8* mRNA expression were investigated; however, they were found not to be upregulated following PSS and PL treatment (Fig. 7I and J). As apoptotic cascade consists of several sequential steps [83]. *BAX* and *CASP8* may not be the effector proteins involved in PSS- and PL-stimulate apoptosis, but other genes such as BCL-2, other caspases, PARP, FAS, or TRAIL [84] should be employed to observe whether they have a potential to this process.

**Fig. 8:**
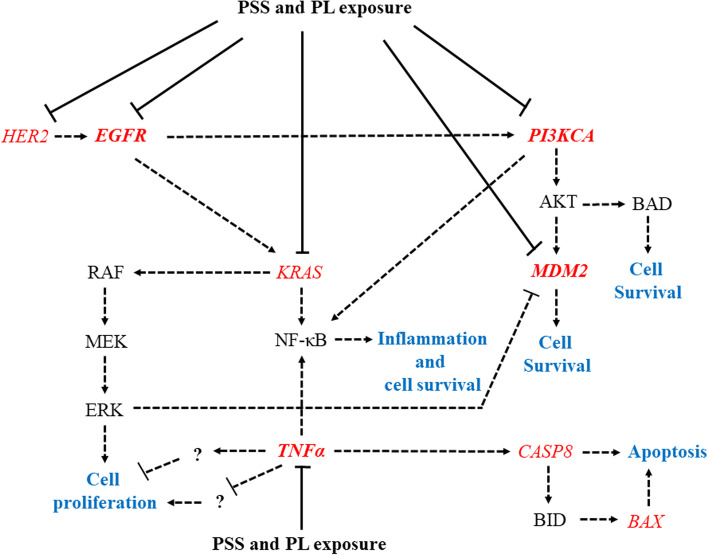
Possible mechanisms responsible for PSS and/or PL treatment in BC cell lines. *HER2, EGFR, MDM2, TNFα, PI3KCA, KRAS, BAX, and CASP8* (red letters) were observed in mRNA expression, they are related to cell survival, cell proliferation, apoptosis, or inflammation process (blue letters). *EGFR*, *MDM2*, *TNFα, and PI3KCA* revealed as common targeted genes of PSS and PL exposure shown in bold red letter. Moreover, the cascading genes of *TNFα* in BC growth suppression should be explored. Solid line with blocked head represents the directly downregulated genes in PSS and/or PL treatment, dotted line represents feasible gene signaling pathway, arrowhead and blocked head means stimulation and suppression, respectively

Concerning breast carcinogenesis, various signaling pathways are known to be involved. In view of this, the present study also explored the mRNA expression levels of genes responsible for BC. Upregulation of a tumor suppressor gene, *BRCA1*, was not identified after PSS and PL exposure (Fig. 7B). A previous study showed that the negativity influence of *BRCA1* expression is associated with ER-/PR-/HER- receptors [85], suggesting that *BRCA1* mRNA expression cannot be used as a follow-up marker for PSS and PL medication in TNBC, and that other genes involved in DNA repair may be responsive to this treatment instead of *BRCA1*. In spite of the negative results identified through an exploration of *BRCA1*, the anti-BC effects mediated through exposure to PSS and PL can be compromised by other genes, as shown in Fig. 7C-H. Both PSS and PL were found to reduce the expression levels of the oncogenic *HER2* gene in all observed BC cell lines (Fig. 7C). Furthermore, HER2-positive patients have been shown to respond better to treatment compared with HER-2-negative patients [86]. Downregulation of *HER2* also accords with that of another ErbB gene family member, *EGFR* (Fig. 7D). Moreover, *HER2* amplification was found to be a cause of breast tumorigenesis through activation of PI3K/Akt pathway, in which *PI3K* overexpression brings about endocrine resistance in BC [87, 88]. According to the findings of the present study, PSS and PL were able to eliminate *PI3KCA* expression (Fig. 7G); this effect has not only been observed at the mRNA level, but additional evidence revealed that the cumulative action of PL or *Smilax* can suppress the phosphorylation of PI3K [89, 90]. PI3K is also an effector signaling molecule downstream of KRAS, and silencing *KRAS* was shown to inhibit PI3K-Akt-mTOR activation, leading to blockade EMT and BC proliferation [40, 91]. We also found that PSS and PL led to a downregulation of *KRAS* (Fig. 7H). *MDM2* expression levels were also detected. The overexpression of *MDM2* was shown to downregulate *TP53,* resulting in human malignancy and chemotherapeutic resistance [92, 93]. Our results revealed that *TP53* was upregulated only in PL-treated MCF7 cells and PSS-treated T47D cells; however, *MDM2* was downregulated in the four tested BC cell lines following exposure to PSS and PL (Fig. 7E). Upregulation of *TP53* may not have been associated with *MDM2* downregulation in every case of PSS or PL treatment due to the fact that breast carcinogenesis is a multistep process involving interactions among multiple gene networks [94]. As described above, PSS and PL participate in cell proliferation and apoptosis, and *TNFα* was previously investigated in view of its dual function in BC, both in cell proliferation or apoptosis [95]. However, a previous study reported that TNFα can induce apoptosis by binding with CASP8 [96, 97]; these findings were not replicated in the present study, which identified a deterioration of the levels of *TNFα* and *CASP8* following PSS and PL exposure (Fig. 7F and J). It may be hypothesized that downregulation of *TNFα* by PSS and PL might involve in the blockage of cell proliferation, and the underlying mechanism should be investigated in greater depth. A scheme of the possible gene networks regulated by PSS and/or PL is shown in Fig. 8.

## Conclusion

The present study has shown that treatment with PSS or PL alone exerted distinctive effects on the suppression of cell growth and colony formation, as well as the induction of apoptosis in BC cell lines, in which *EGFR*, *MDM2*, *TNFα, and PI3KCA* have been clearly demonstrated to be common targets of PSS and PL. However, PSS at low dose contributed to these results in comparison to PL, which has to be used at high dose, assuming that PSS has a synergistic effect on breast cancer cell suppression. In our future studies, PSS and PL will be further investigated in an animal model and in a clinical trial study to identify other risk factors, in order to clearly understand the underlying pathway of action and to establish guidelines for adjuvant herbal dug use of BC prevention.

## Supplementary Information

**Additional file 1: **Supplementary Figures.

## Data Availability

The dataset used and/or analyzed during the current study are available from the corresponding author on reasonable request.

## Abbreviations

PSS: Mixture extract of *P.linteus*, *S. corbularia* and *S. glabra*
PL: Sole *P.linteus* extract
SC: *S. corbularia*
SG: *S. glabra*
