# Supplementary material for: Effect of Smilax spp. and Phellinus linteus combination on cytotoxicity and cell proliferation of breast cancer cells
Source: BMC Complement Med Ther. 2023 Jun 1;23:177. doi: 10.1186/s12906-023-04003-x (PMC10233913; doi:10.1186/s12906-023-04003-x)
Supplement: Supplementary file 1 — Additional file 1: Supplementary Figures. [file 12906_2023_4003_MOESM1_ESM.docx]

**SUPPLEMENTARY**

**Supplementary Figure**

**
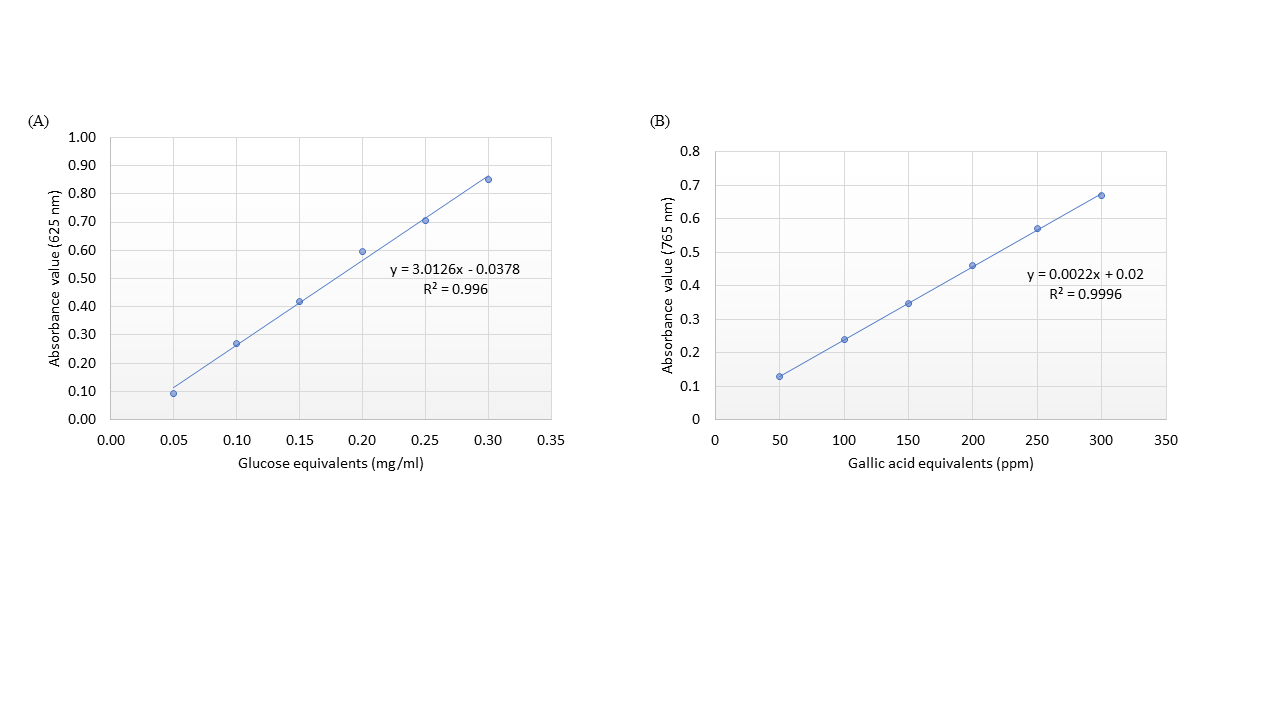
**

**Supplementary Fig.1** Determination of polysaccharide (A) and total phenolic compound (B). The anthrone [44] and Folin-Ciocalteu’s phenol reagent [45] were used to generate the standard curves.

**
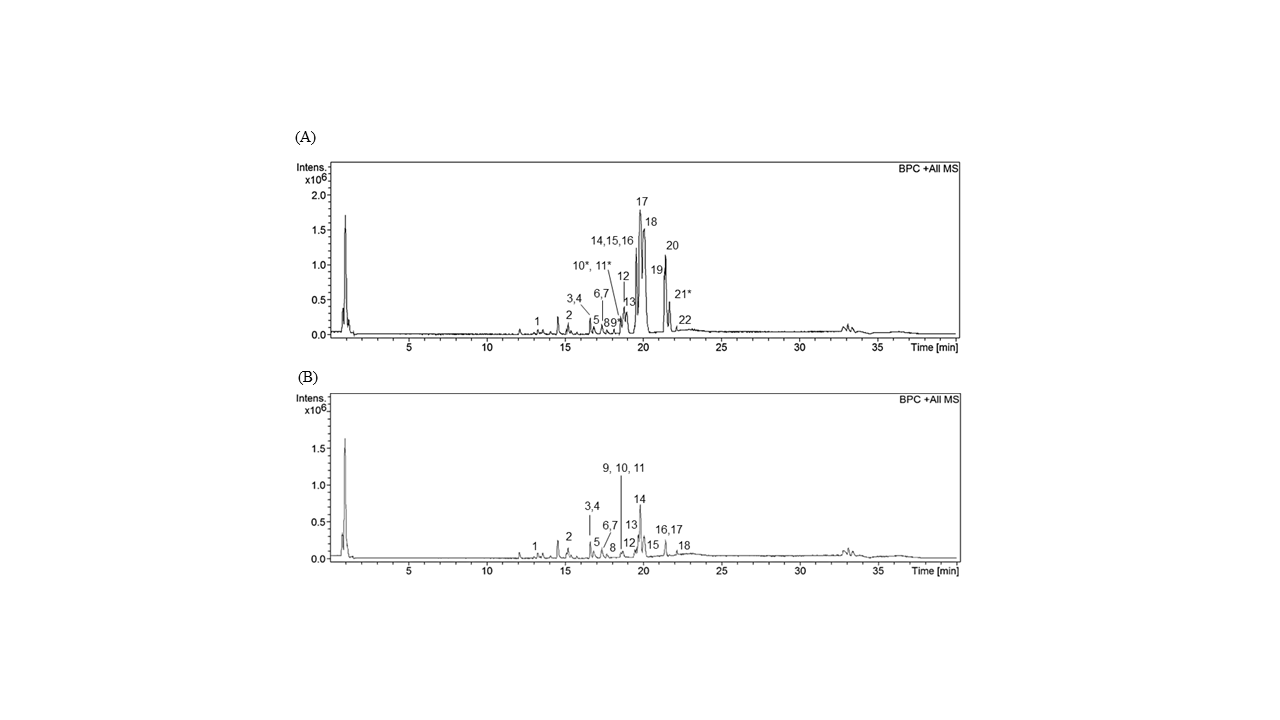
**

**Supplementary Fig.2**

LC/QTOF-MS chromatogram of the PSS extract (A), and the PL extract (B)

**
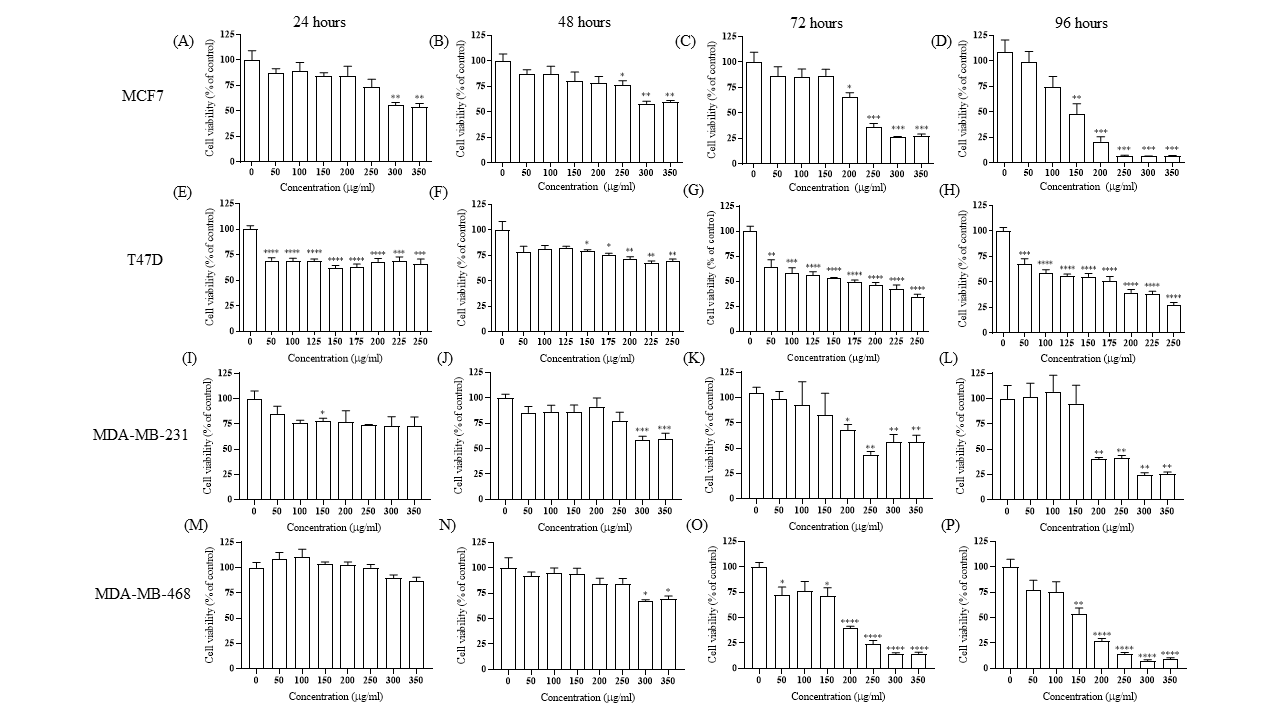
**

**Supplementary Fig. 3** Effect of PSS exposure on 4 breast cancer cell lines in dose- and time- dependent manners. (A-D) MCF7, (E-H) T47D, (I-L) MDA-MB-231, and (M-P) MDA-MB-468. Graph were plotted from 5 replicates for each dose. Error bar denoted as SE, and ^*^, ^**^, ^***^, ^****^ represented P≤0.05, P≤0.01, P≤0.001, and P≤0.0001 from statistical analysis using Unpaired t-test compared between the untreated cells among the same group.

**
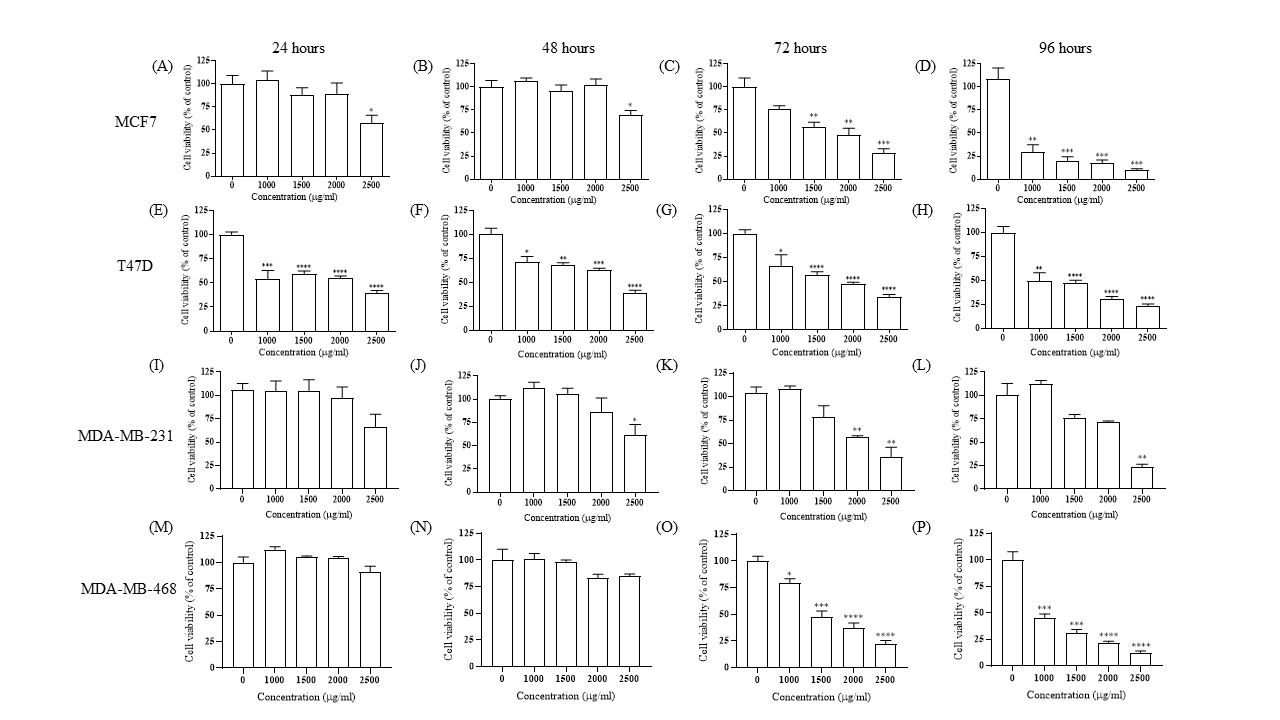
**

**Supplementary Fig. 4** Effect of PL exposure on 4 breast cancer cell lines in dose- and time- dependent manners. (A-D) MCF7, (E-H) T47D, (I-L) MDA-MB-231, and (M-P) MDA-MB-468. Graph were plotted from 5 replicates for each dose. Error bar denoted as SE, and ^*^, ^**^, ^***^, ^****^ represented P≤0.05, P≤0.01, P≤0.001, and P≤0.0001 from statistical analysis using Unpaired t-test compared between the untreated cells among the same group.

**
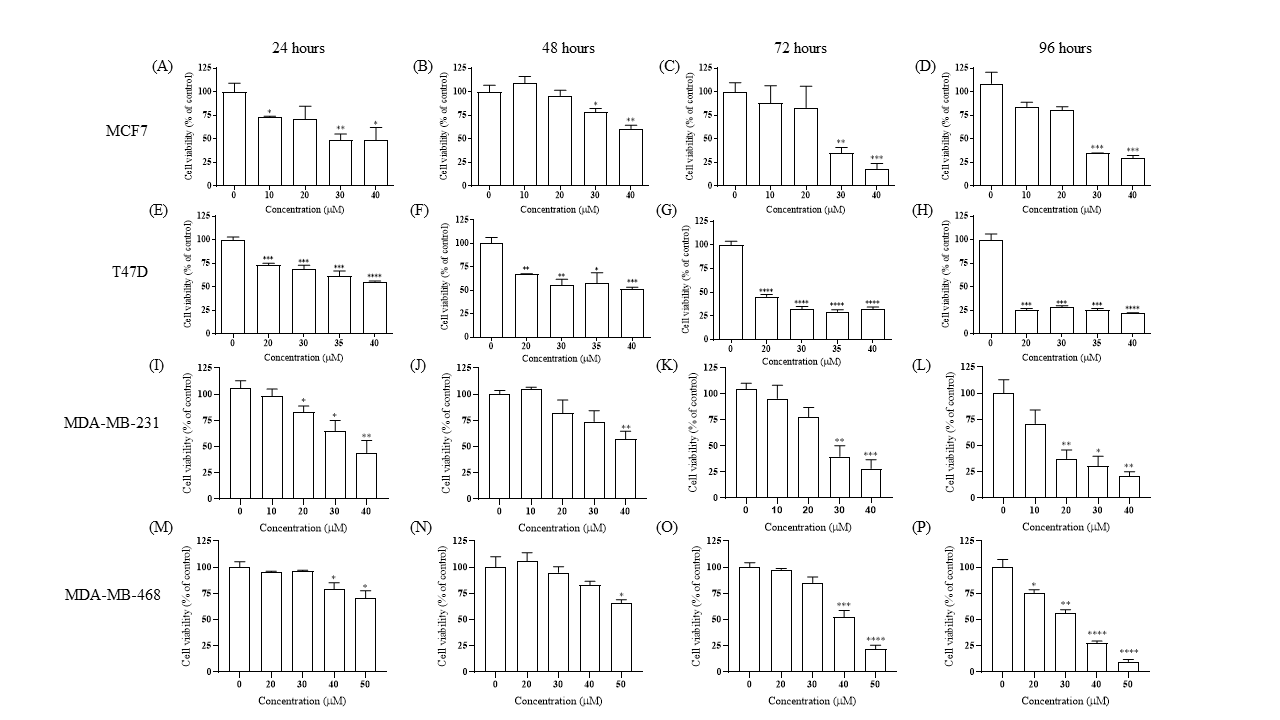
**

**Supplementary Fig. 5** Effect of Cisplatin treatment on 4 breast cancer cell lines in dose- and time- dependent manners. (A-D) MCF7, (E-H) T47D, (I-L) MDA-MB-231, and (M-P) MDA-MB-468. Graph were plotted from 5 replicates for each dose. Error bar denoted as SE, and ^*^, ^**^, ^***^, ^****^ represented P≤0.05, P≤0.01, P≤0.001, and P≤0.0001 from statistical analysis using Unpaired t-test compared between the untreated cells among the same group.

**
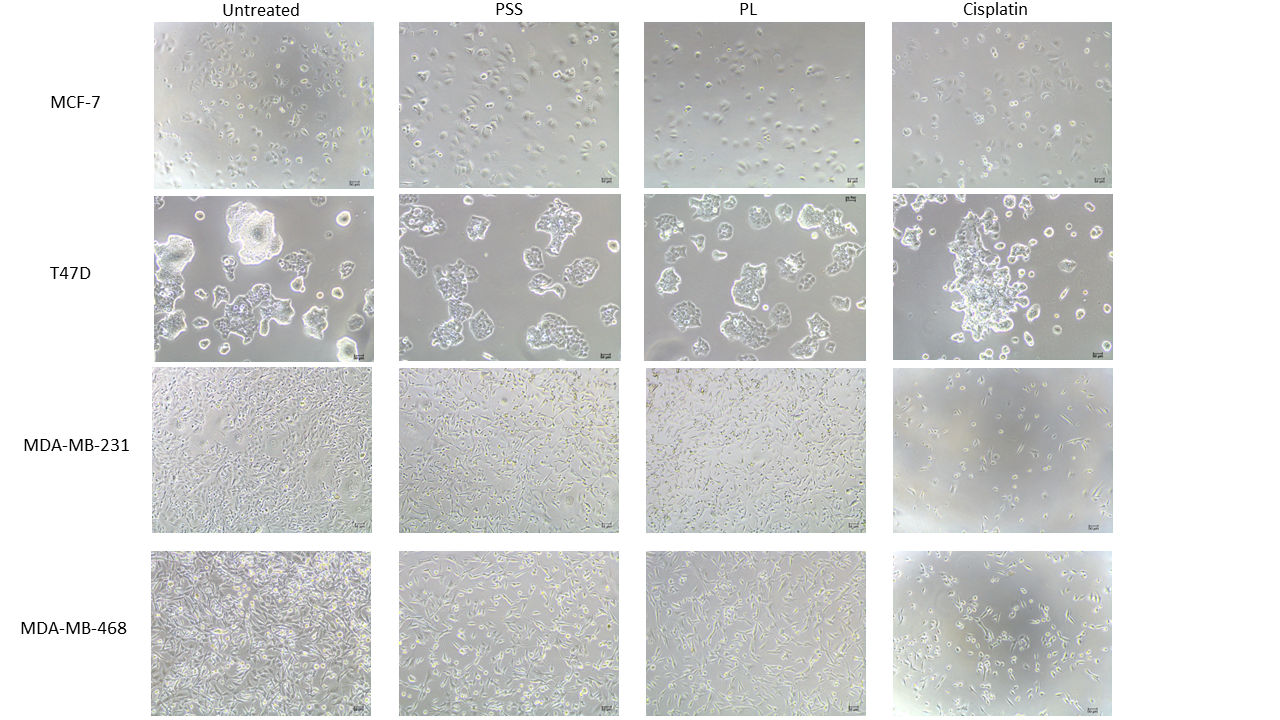
**

**Supplementary Fig. 6** Breast cancer cell morphology after PSS, PL, and Cisplatin treatments for 3 days.


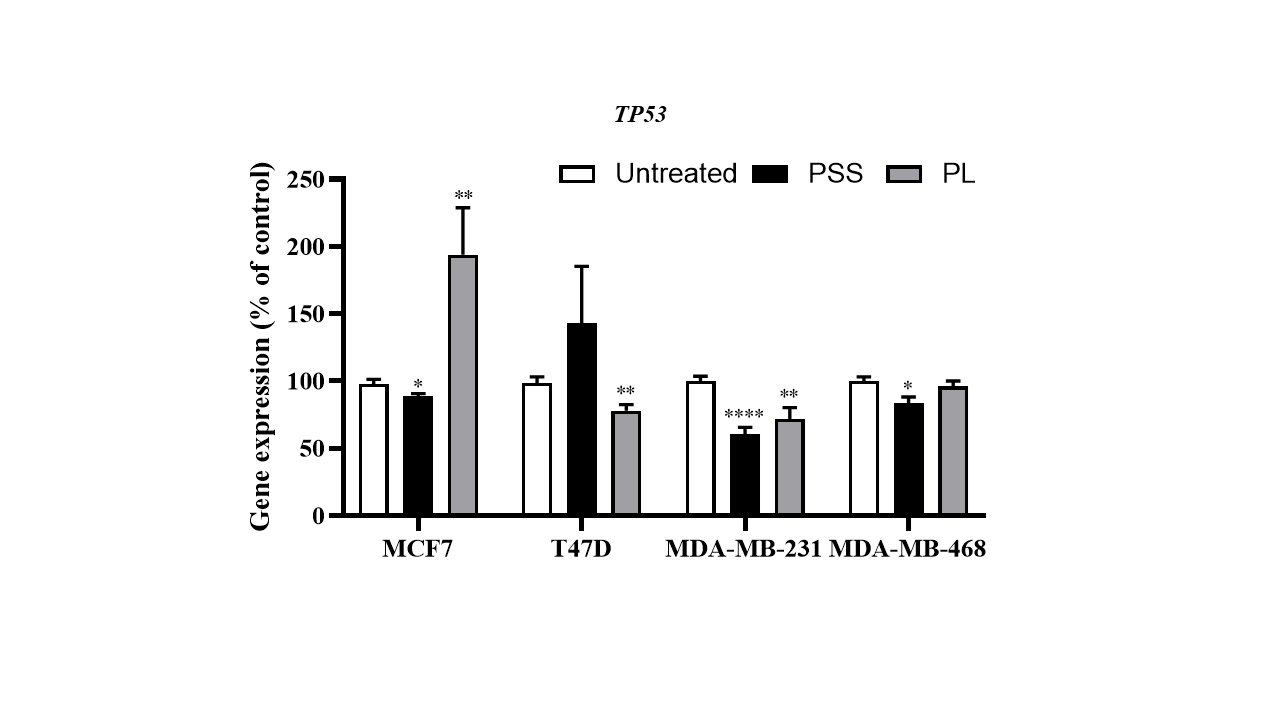


**Supplementary Fig. 7** The expression changes of *TP53* in mRNA level in 4 breast cancer cell lines after PSS and PL treatment. Data represents 4 independent replicates as mean ± SE; ^*^, ^**^, ^****^ represented P≤0.05, P≤0.01, and P≤0.0001 from statistical analysis using Unpaired t-test compared between the untreated cells among the same group.
